# Systemic and airway T cell dynamics with influenza-specific immune recovery by cystic fibrosis elexacaftor/tezacaftor/ivacaftor therapy

**DOI:** 10.1186/s12931-026-03521-9

**Published:** 2026-01-22

**Authors:** Elli Mouchtaridi, Aleksandra Kowalik, Elisa J. M. Raineri, Marion Humbert, Josef Jägerstedt, Margaret Bojarlind, Kristina Nilsson, Malin Flodström-Tullberg, Terezia Pincikova, Johan K. Sandberg

**Affiliations:** 1 Center for Infectious Medicine, Department of Medicine, Karolinska Institutet, Stockholm, Sweden; 2 Division of Pediatrics, Department of Clinical Science, Intervention and Technology, Karolinska Institutet, Stockholm, Sweden; 3 Stockholm CF Center, Karolinska University Hospital Huddinge, Stockholm, Sweden

**Keywords:** Cystic fibrosis, T cells, Influenza A virus, Sputum, Blood, Inflammation, MAIT cells, Elexacaftor, Tezacaftor, Ivacaftor

## Abstract

**Background:**

Therapy with elexacaftor/tezacaftor/ivacaftor (ETI) works to improve the functionality of the cystic fibrosis (CF) transmembrane conductance regulator (CFTR) protein and has revolutionized CF treatment. However, the implications of ETI for airway barrier and systemic T cell immunobiology remain relatively little studied.

**Methods:**

Here, we investigated the immunological effects of ETI at systemic and local pulmonary levels, using paired peripheral blood and sputum sampling, in relation to key clinical parameters. Samples were taken longitudinally at baseline (*n* = 27), and at three (*n* = 24) and 12 months (*n* = 19) of treatment and subjected to analysis by advanced flow cytometry, T cell assays, and plasma proteomics.

**Results:**

Before ETI treatment initiation, immune cell composition in the sputum closely reflected the plasma inflammatory proteome. T cell abundance in sputum correlated inversely with multiple plasma factors, including IL-17A, IL-8, HGF and TGFα, and with lower sweat chloride concentrations. Chronic microbial infection was associated with low abundance of CD4 T cells and mucosa-associated invariant T (MAIT) cells in sputum samples collected at baseline. During ETI treatment, T cells with lung resident characteristics including MAIT cells increased in sputum, accompanied by improved lung function and reduced systemic inflammation. In peripheral blood, the effector-memory CD8 and CD4 T cell pool expanded and the magnitude and quality of T cell responses to Influenza A virus recovered during ETI.

**Conclusions:**

These findings indicate that ETI treatment promotes immunological remodelling in both airways and circulation, correlating with favorable changes in clinically relevant parameters, and a shift towards healthy immune regulation in the lung and improved adaptive T cell responses in circulation.

**Supplementary Information:**

The online version contains supplementary material available at 10.1186/s12931-026-03521-9.

## Introduction

Cystic fibrosis (CF) is the most common life-shortening autosomal recessive genetic condition [1]. Cystic fibrosis is caused by mutations in the CF transmembrane conductance regulator (*CFTR*) gene, which encodes the CFTR chloride channel expressed on the apical surface of epithelial cells, leading to impaired ion transport and abnormally thick secretions [2]. Due to the broad tissue distribution of CFTR, multiple epithelial organs and mucosal surfaces are affected and compromised. The most common cause of morbidity and mortality in CF is progressive lung disease driven by a vicious circle of airway infection and inflammation.

Components of the immune system are critical for maintenance of pulmonary barrier homeostasis and for the clearance of pathogens and pollutants without excessive inflammation [3, 4]. CFTR dysfunction impairs mucociliary clearance in the airways, leading to compromised barrier function, mucus retention, acute and chronic airway infections and exaggerated neutrophil-dominated inflammation [5, 6]. This inflammatory milieu is characterized by high levels of cytokines such as IL-6, IL-8, IL-1β, and TNF, attracting hyperactive and functionally impaired neutrophils that contribute to tissue damage and persistent infections [7]. Over time, this causes decline in pulmonary function, irreversible lung damage and eventually, respiratory insufficiency. CFTR-deficient epithelial cells exhibit elevated basal pro-inflammatory signaling, intrinsic cell stress responses, and apoptosis, contributing to dysregulated innate barrier immunity [8]. Additionally, the adaptive immune response is impaired, with suppressed T cell activity in the inflamed CF lung [9, 10]. Dominant Th2 and Th17 responses contribute to immunopathogenesis and inflammation via IL-4, IL-13, and IL-17A, with ineffective control of CF-associated pathogens such as *Pseudomonas aeruginosa* and *Aspergillus spp*. [11–16]. Furthermore, respiratory viral infections such as respiratory syncytial virus and influenza virus are common initiators of lung exacerbations in CF [17, 18].

CFTR modulator therapies enhance the function of mutant CFTR proteins by improving processing, trafficking, and channel activity. The combination of ivacaftor, a potentiator of CFTR function, and lumacaftor, a corrector of CFTR misfolding, demonstrated modest improvements in lung function in F508del homozygotes [19]. More recently, the highly effective modulator therapy with triple combination of elexacaftor, tezacaftor, and ivacaftor (ETI), designed for people with cystic fibrosis (pwCF) with at least one copy of the F508del *CFTR* variant, has revolutionized CF care by significantly improving lung function, quality of life, and reducing pulmonary exacerbations [20, 21]. With chronic inflammation having broad consequences for health, the impact of ETI on systemic inflammation and the function of the immune system will be important for the long-term health status of pwCF [22]. Early studies have indicated that various soluble and cellular mediators of inflammation improve in response to ETI [23–26], along with consistent but incomplete restoration of control of pulmonary infections [27]. However, a deeper understanding of the impact of ETI on the function of the immune system, including adaptive T cell immunity, is needed to fully understand the long-term effects of this treatment.

In this real-world observational study with longitudinal sampling of both sputum and peripheral blood, we provide a comprehensive characterization of the impact of ETI on the immune system in the airways and systemic circulation, in relation to key clinical parameters in pwCF. Our results indicate that the immune cell composition in sputum at baseline closely reflects disease state and systemic inflammation, with higher abundance of T cells in sputum associated with better lung function, lower sweat chloride concentrations and lower inflammatory mediators in plasma. Increased T cell abundance in sputum during ETI treatment was accompanied by decreased granulocyte levels, improved lung function, and reduced systemic inflammation. In blood, T cell compartment characteristics and the quality of T cell responses to Influenza A virus (IAV) were enhanced during ETI treatment. These findings suggest that ETI treatment promotes beneficial adaptive immunological remodelling primarily in the airways of pwCF that may contribute to improved clinical outcomes through recovery of T cell immune homeostasis.

## Methods

### Sex as a biological variable

Samples from both male and female pwCF were included in this study.

### Study subjects and sampling

The study participants were part of the Stockholm ETI Task Force Study cohort. Briefly, the Stockholm ETI Task Force Study is an observational cohort study designed to investigate the immunobiological and clinical beneficial effects and side effects of ETI treatment in a real-world setting in pwCF followed at the Stockholm CF Center. The participants (*n* = 27) were recruited when initiating ETI therapy between 2023–02-01 and 2023–12–31 while in stable condition. They underwent assessments at baseline, and at three and 12 months following initiation of ETI treatment, in accordance with the Swedish national ETI follow-up program. The study participants were in clinically stable condition at all visits. Clinical characteristics of the study participants at the baseline timepoint are shown in Table 1.

**Table 1:** Clinical and laboratory characteristics of the study cohort at the ETI treatment initiation visit

Characteristics	
Female/Male, n (%)	10 (37)/17 (63)
Age, median (range), years	32 (14–73)
Genotype, F508del homozygote, n (%)	12 (44)
Genotype, F508del heterozygote, n (%)	14 (52)
Genotype, other, n (%)	1 (4)
Sweat chloride (mmol chloride/L)	80 (38)
FEV_1_, % predicted	83 (26)
FVC, % predicted	95 (28)
BMI, kg/m^2^, median (range)	23.8 (16.3–38.5)
Pancreatic insufficiency, n (%)	19 (70)
Diabetes, n (%)	6 (22)
Chronic infection with *Pa*, n (%)	8 (30)
Chronic infection with *Sa*, n (%)	7 (26)
CRP (mg/L)	1 (1)
ESR (mm)	7 (18)
Leucocyte count (× 10^9^/L)	6.1 (2.1)
Neutrophil count (× 10^9^/L)	3.3 (1.6)
Platelet count (× 10^9^/L)	293 (102)
IgG (g/L)	11 (2.8)
Albumin (g/L)	38 (5)
Hemoglobin (g/L)	147 (21)
HbA1c (mmol/mol)	37 (6)
Glucose (mg/dL)	5.9 (2.1)

*ETI* Elexacaftor/tezacaftor/ivacaftor*, FEV*_*1*_ forced expiratory volume in 1 s, *FVC* forced vital capacity*, BMI* body mass index*, Pa Pseudomonas aeruginosa*, *Sa Staphylococcus aureus, CRP* C-reactive protein*, ESR* erythrocyte sedimentation rate*. Values are medians with interquartile ranges, unless stated otherwise*

Twelve study subjects were homozygous for the F508del *CFTR* variant and had switched from lumacaftor/ivacaftor treatment to ETI. The remaining 15 study subjects had no previous CFTR modulator treatment. One subject had *CFTR* variants other than F508del. Blood and sputum samples were collected at the baseline, 3-month and 12-month visits. Sputum could not always be collected. Number of biological samples available for the different analyses are stated in the relevant figure legends. A total of seven patients discontinued ETI treatment during the 12-month period due to various side effects such as rash, elevated transaminases, lymphadenopathy, headache, or lack of adherence. During follow-up, one patient developed allergic bronchopulmonary aspergillosis, requiring systemic corticosteroids and antifungal therapy. Three patients experienced severe pulmonary exacerbations between six and 11 months after initiation of ETI, all of which necessitated treatment with intravenous antibiotics.

The clinical parameters recorded in the study included sweat chloride measurement by Macroduct Advanced Sweat Collection System (ELITech), the body mass index (BMI), sputum culture for bacteria, fungi and mycobacteria, and inflammatory parameters measured using in-house methods at the Karolinska University Hospital laboratory including complete blood count with differential leucocyte count, erythrocyte sedimentation rate (ESR), C-reactive protein (CRP), and serum total IgG. To assess lung function, dynamic spirometry was performed and forced expiratory volume in 1 s (FEV₁) percent predicted values were calculated using the Solymar reference equations for individuals with CF under 18 years of age, and the Hedenström reference equations for those aged 18 years and older. Additional sputum and blood samples were collected for analysis by flow cytometry and for plasma proteomics.

Chronic infection was defined as repeated sputum cultures testing positive for any pathogenic bacterial, mycobacterial, or fungal agents within the 12 months preceding the initiation of ETI treatment. Identified pathogens included *Pseudomonas aeruginosa, Staphylococcus aureus, Mycobacterium avium, Mycobacterium abscessus, Mycobacterium lentiflavum, Stenotrophomonas maltophilia* and *Aspergillus fumigatus*.

### Sample collection

The majority of the collected sputum samples were induced during physiotherapy sessions by a trained pulmonary CF physiotherapist following a standardized sputum induction protocol. Following an inhaled bronchodilator, subjects inhaled hypertonic saline (4–7%) via a mesh nebulizer (eFlow Rapid, PARI Pharma GmbH, Germany) to loosen airway secretions. Airway clearance techniques (positive airway pressure, autogenic drainage, forced expiration techniques) were performed to mobilize and evacuate secretions. The expectorated sputum was collected in a standard sputum container. Peripheral blood samples were obtained in sodium-heparin tubes from a peripheral vein or a subcutaneous venous port.

### Blood processing and cell isolation

Peripheral blood mononuclear cells (PBMCs) were isolated from whole blood after ficoll density separation gradient (Cytiva). After isolation, PBMCs were frozen in DMSO (Sigma-Aldrich) supplemented with 10% FCS (Thermo Fisher Scientific) and stored in −120 °C until further use. Plasma samples were collected after spinning whole blood at 1900 rpm for 10 min and stored at −80 °C.

### Sputum cell isolation

Sputum samples were collected and immediately processed. Sputolysin reagent (Merck) was used in equal sample volume, and the mix was incubated for 15 min on a shaker at 37 °C. RPMI was used afterwards to wash, and the mix was passed through 70 μM filters. Cells were counted and stained for flow cytometry immediately.

### Flow cytometry

Sputum sample cells were stained immediately after isolation, while PBMCs were thawed and rested for 3 h at 37 °C in RPMI 1640 (Cytivia) supplemented with 2 mM L-Glutamine (Cytivia), 10% FCS (Sigma), HEPES (Gibco), 50 μg/mL gentamicin (Gibco) and 100 μg/mL normocin (Invivogen) in a concentration of 1 × 10^6^ cells per well in 96-well U-bottom plates (Corning). Monoclonal antibodies for cell surface and intracellular staining are listed in Supplemental Table 1 and Supplemental Table 2, respectively. Cells were plated in 96-well plates and incubated with Fc-block (Merck) for 10 min at room temperature. For identification of MAIT cells, hMR1-5-O*P-*RU tetramer (NIH tetramer Core facility) staining was performed for 30 min at 4 °C. Chemokine receptor staining was performed for 10 min at 37 °C, whereas all other cell surface staining was performed at 4 °C for 20 min, and cells were next washed with PBS containing 2% FCS and 2 mM EDTA. Intracellular and transcription factor staining was performed using the BD Fixation/Permeabilization Kit (BD Biosciences) or the Foxp3/Transcription Factor Staining Buffer Set (eBioscience), respectively. Samples were acquired on a BD Symphony A5 instrument (BD Biosciences) equipped with 355 nm, 405 nm, 488 nm, 561 nm, and 639 nm lasers. Single-stained polystyrene beads (BD Biosciences) were used for compensation, which was performed using the compensation platform in FlowJo software v. 9.8 (Tree Star). For polyfunctionality analysis the SPICE software v6.1 was used [28].

**Table 2:** Comparison of subgroups stratified by prior treatment with lumacaftor/ivacaftor, based on data collected at the ETI treatment initiation visit

Characteristics	Never taking CFTR modulators (*n* = 15)	Switched from LUM/IVA to ETI (*n* = 12)	*p-*value
Female/Male, n (%)	4 (27)/11 (73)	6 (50)/6 (50)	0.212^a)^
Age, median (range), years	37 (14–73)	26 (15–53)	**0.043**^b)^
Sweat chloride (mmol chloride/L)	64 (48)	83 (21.5)	0.441^b)^
FEV_1_, % predicted	87 (22)	85 (30)	0.790^b)^
FVC, % predicted	94 (23)	101 (21)	0.346^b)^
BMI, kg/m^2^, median (range)	24.2 (19.9–38.5)	23.3 (16.3–28.2)	0.110^b)^
Pancreatic insufficiency, n (%)	7 (47)	12 (100)	N/A^a)^
Diabetes, n (%)	3 (20)	3 (25)	0.756^a)^
Chronic infection *Pa*, n (%)	3 (20)	5 (42)	0.221^a)^
Chronic infection *Sa*, n (%)	2 (13)	5 (42)	0.095^a)^
CRP (mg/L)	1 (1)	1 (2)	0.784^c)^
ESR (mm)	7 (22)	5 (11)	0.894^c)^
Leucocyte count (× 10^9^/L)	6.1 (1.3)	6 (2.6)	0.873^b)^
Neutrophil count (× 10^9^/L)	3.6 (1.5)	3.2 (2.6)	0.743^b)^
Platelets count (× 10^9^/L)	282 (102)	306 (146)	0.214^b)^
IgG (g/L)	11.9 (2.7)	10.5 (3.3)	**0.021**^c)^
Albumin (g/L)	37 (5)	39 (5)	0.486^b)^
Hemoglobin (g/L)	147 (22)	145 (25)	0.666^b)^
HbA1c (mmol/mol)	37 (6)	38 (7)	0.453^c)^
Glucose (mg/dL)	5.9 (2.1)	5.9 (2.7)	0.830^c)^

*ETI* Elexacaftor/tezacaftor/ivacaftor; *LUM/IVA* Lumacaftor/ivacaftor; *FEV*_*1*_ forced expiratory volume in 1 s, *FVC* forced vital capacity*, BMI* body mass index*, Pa Pseudomonas aeruginosa*, *Sa Staphylococcus aureus, CRP* C-reactive protein*, ESR* erythrocyte sedimentation rate*. Values are medians with interquartile ranges, unless stated otherwise. p-value*

^*a)*^*Chi-square*

^*b)*^*t-test*

^*c)*^*Mann–Whitney test*

### Peptide antigen

The PepTivator® Influenza A (H1N1) NP peptide pool is a mix of 15-mers with 11 amino acids overlap spanning the nucleoprotein (Miltenyi Biotec).

### Activation-Induced Marker assay

PBMCs were thawed, resuspended in RPMI 1640 (Cytivia) supplemented with 2 mM L-Glutamine (Cytivia), 10% FCS (Sigma), HEPES (Gibco), 50 μg/mL gentamicin (Gibco) and 100 μg/mL normocin (Invivogen) and rested for 10 h at 37 °C in a concentration of 1 × 10^6^ cells per well in 96-well U-bottom plates (Corning). For assessment of Influenza-specific T cell responses, the PepTivator® Influenza A (H1N1) NP peptide pool (1 ug/ml per peptide, Miltenyi Biotec) were added. Anti-CXCR5 antibody (BD Biosciences) was added in the medium 15 min before the peptide pool. Negative control wells contained only medium. Positive control wells included 0.1 μg/ml Staphylococcal enterotoxin B (SEB) (SIGMA). After 1 h, GolgiPlug and GolgiSTOP (BD Biosciences) were used to block the secretory pathway according to manufacturer’s instructions. Cells were incubated at 37 °C for a total of 11 h, and then washed with PBS containing 2% FCS and 2 mM EDTA and stained with anti-CCR6-BUV737 and anti-CCR7-APC-Cy7 for 10 min at 37 °C. Cells were further surface stained for 20 min at 4 °C and stained intracellularly for 30 min at 4 °C using the Fixation/Permeabilization buffer kit (BD Biosciences). Stimulation index was defined as the fold-change between peptide-stimulated and unstimulated samples. Samples with stimulation index > 1.5 were considered as responders and the percentage of those responders were depicted in the corresponding figures. The specific CD4 and CD8 T cell response shown in Fig. 4, B and D is the adjusted response, calculated after subtraction of the background in unstimulated samples.

### Pseudomonas aeruginosa preparation and cell stimulation

*P. aeruginosa* (ATCC 27853) working stocks and formaldehyde fixation were performed as previously described [29]. For the stimulation experiments, PBMCs were thawed and rested for 3 h at 37 °C in RPMI media at a concentration of 1 × 10^6^ cells per well in 96-well U-bottom plates before mildly formaldehyde-fixed *P. aeruginosa* were added at a microbial dose of 50:1 and incubated for 24 h. GolgiPlug and GolgiSTOP (BD Biosciences) were added after 18 h of stimulation.

### Proximity extension assay proteomics

Proximity extension assay technology was used to compare the soluble proteome in plasma of individuals with CF at baseline (*n* = 27), three months (*n* = 24) and 12 months (*n* = 19). One baseline sample that did not pass quality control was not included in further analysis. Out of 92 targets, 73 were used for further analysis (targets not detected in ≥ 50% of samples were excluded). Samples were analyzed in one batch using the Olink Target 96 Inflammation Panel (Olink Proteomics). Data generation was performed by the Affinity Proteomics Stockholm Unit at the Science for Life Laboratory, Stockholm, Sweden. Differential expression analysis was performed using the Wilcoxon rank sum test with Benjamini–Hochberg correction implemented in the olink_wilcox function in the OlinkAnalyze package (version 4.2.0). Correlations were calculated with the cor.test function from the statspackage (version 4.4.3). Data visualization was performed using ggplot2 (version 3.5.2) and pheatmap (version 1.0.12). All analyses were conducted in R (version 4.4.3).

### Statistical analysis

The Wilcoxon Signed Rank test was used for comparison of non-parametrically distributed paired data. For comparison of unpaired data, the t-test and Mann–Whitney test were used. The Spearman rank correlation test was used to compare correlations between two parameters. Spearman correlations between clinical characteristics and flow cytometry cell data were calculated independently for each parameter, using all patients with available data for each specific measure. Δ values for clinical, flow cytometry and NPX data were calculated as paired differences (3 M-0 M and 12 M-0 M). Statistical analyses were performed with GraphPad Prism v.6.0c (GraphPad Software). Where SPICE was used, permutation test was performed to compare the different overlays. *P-*values < 0.05 were considered significant.

## Results

### Study cohort characteristics

**Fig. 1:**
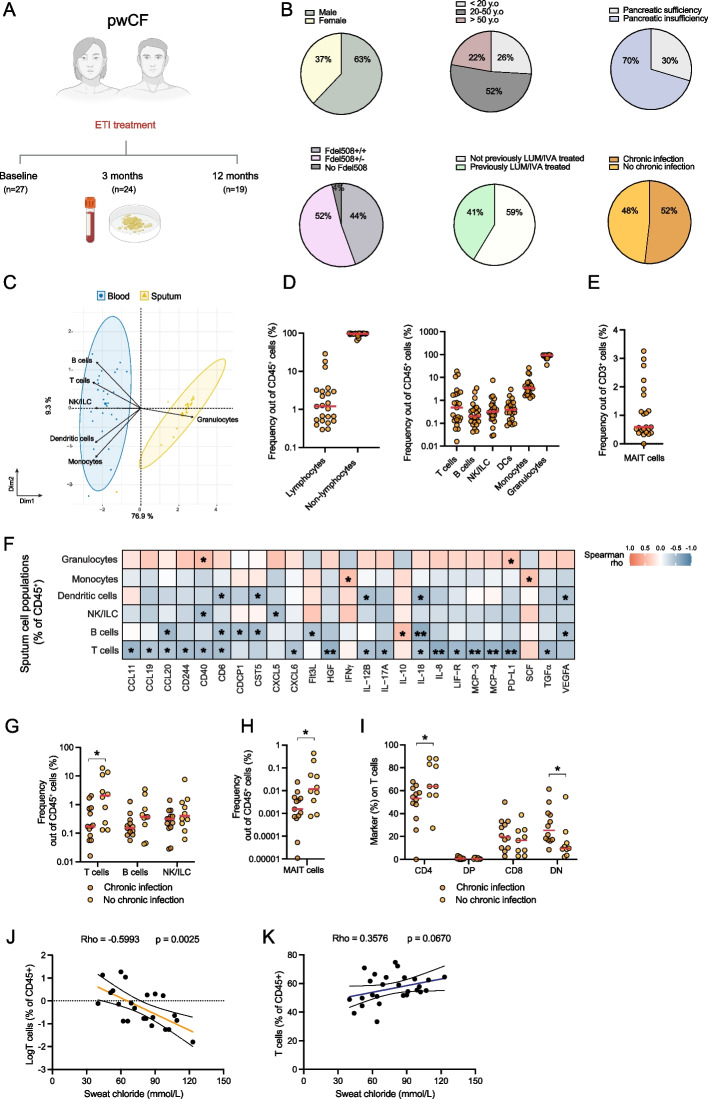
Cohort overview and sputum immune cell composition in people with cystic fibrosis at baseline. **A** Schematic overview of the study design depicting blood and sputum sample collection at baseline, 3 months and 12 months after starting ETI treatment. **B** Pie charts depicting the sex (top left), age (top middle), pancreatic insufficiency (top right), presence of Fdel508 CFTR variant (bottom left), previous treatment with lumacaftor/ivacaftor (LUM/IVA) (bottom middle) and presence of chronic airway infection (bottom right) in pwCF study participants (*n* = 27). **C** PCA plot showing cellular distribution of paired blood (*n* = 27) and sputum (*n* = 23) samples at baseline. **D** Scatter dot plot showing the main immune cell populations as percentages out of live CD45^+^ cells from sputum at baseline (*n* = 23). Scale is logarithmic. **E** Scatter dot plot showing the percentage of MAIT cells out of total T cells measured in the sputum of pwCF at baseline (*n* = 23). **F** Heatmap showing Spearman correlations between the sputum cell populations identified by flow cytometry and plasma inflammatory proteins at baseline (*p < 0.05, **p < 0.01, ***p < 0.001) (*n* = 22). **G** Scatter dot plot showing the lymphocyte populations in sputum in pwCF that had chronic airway infection at baseline (Kruskal–Wallis, *p < 0.05, **p < 0.01) (*n* = 23). Scale is logarithmic. **H** Scatter dot plot showing the MAIT cell percentages in sputum that had a positive or negative sputum culture at baseline (Kruskal–Wallis, *p < 0.05, **p < 0.01) (*n* = 23). Scale is logarithmic. **I** The CD4 and CD8 T cell composition in sputum in pwCF that had chronic airway infection at baseline (Kruskal–Wallis, *p < 0.05, **p < 0.01) (*n* = 21). **J** Spearman correlation plots between total T cell percentages in the sputum and F sweat chloride concentration (mmol/L) at baseline (*n* = 23). Scale is logarithmic. **K** Spearman correlation plots between total T cell percentages in the blood and sweat chloride concentration (mmol/L) at baseline (*n* = 27). Red line represents median in panels (**D-I**)

To study the impact of ETI on immune status and associations with clinical parameters, 27 pwCF initiating ETI treatment were recruited and followed within the scope of the observational real-world Stockholm ETI Task Force Study (Table 1). The 27 individuals were sampled for sputum, plasma and PBMCs at baseline with follow-up sampling at 3 months (*n* = 24) and 12 months (*n* = 19) after ETI treatment initiation (Fig. 1A). The median age of the cohort at baseline was 32 years (range: 14–73) and 37% were female. Chronic infection with CF-associated bacterial or fungal pathogens was observed in 52% of the participants at baseline, whereas pancreatic insufficiency was present in 70% of the participants. Twelve individuals (44%) were homozygous for the F508del *CFTR* variant and had been treated with lumacaftor/ivacaftor prior to switching to ETI while the remaining study subjects had no prior CFTR modulator treatment (Fig. 1B, Table 1). Comparative evaluation of the clinical characteristics and laboratory measurements between pwCF with no prior CFTR modulator therapy before ETI initiation (*n* = 15) and those who switched from lumacaftor/ivacaftor to ETI (*n* = 12) revealed no differences at baseline, except for serum IgG levels that were lower in the previously treated group (Table 2).

### T cell abundance in the sputum reflects lower systemic inflammation and better clinical outcomes

Flow cytometry analysis of sputum samples at baseline revealed heterogeneous immune cell content, including both lymphocyte and non-lymphocyte populations (Supplemental Fig. 1A). Granulocytes were the dominant cell type, consistent with the inflammatory profile characteristic of the CF lung environment with the vast majority of those being neutrophils (Fig. 1C and Supplemental Fig. 1B). In addition, major immune cell subsets were detected, including T cells, B cells, natural killer (NK) cells, dendritic cells (DCs), and monocyte-like cells (Fig. 1D and Supplemental Fig. 1C). Within the sputum T cell population, mucosal-associated invariant T (MAIT) cells were identified and their frequencies varied among individuals (Fig. 1E and Supplemental Fig. 1D).

Plasma proteomics using the proximity extension assay technology with the Olink Target 96 Inflammation panel revealed multiple correlations with immune cell composition in the sputum, indicating the local pulmonary immune landscape is reflected in patterns of systemic inflammation (Fig. 1F). Notably, sputum T cell frequencies correlated negatively with 25 out of 73 proteins detected in plasma, suggesting that an abundance of T cells in the CF lung is associated with a less active inflammatory state systemically. Among inflammatory mediators correlating negatively with T cells abundance in sputum were IL-8 and IL-17A. Furthermore, proteins indicative of tissue damage and repair including hepatocyte growth factor (HGF) [30], and transforming growth factor α (TGFα) [31], were negatively associated with T cell abundance in sputum. Moreover, granulocyte frequencies in sputum correlated positively with CD40 and PD-L1 levels (Fig. 1F). In contrast, blood T cell frequencies showed no significant correlation with soluble plasma markers, whereas blood granulocyte frequencies correlated positively with IL-8 levels (Supplemental Fig. 1E).

Chronic infections by bacterial or fungal pathogens were associated with low abundance of T cells (Fig. 1G), and MAIT cells (Fig. 1H), in sputum samples collected at baseline. Furthermore, individuals with chronic infection at baseline had higher frequencies of CD4⁻CD8⁻ (CD4 and CD8 double-negative, DN) T cells within the sputum T cell pool (Fig. 1I), suggesting that reduced abundance of conventional CD4⁺ and CD8⁺ T cells is associated with the presence of chronic infection in the CF airways. Finally, lower sweat chloride concentrations (mmol/L), a key clinically relevant biomarker of CFTR function, were found to correlate with T cell abundance in sputum (Fig. 1J), but not in peripheral blood (Fig. 1K), highlighting the relevance of airway-derived immune profiling in CF. No significant correlation was observed between either FEV_1_ or sweat chloride concentration and age (Supplemental Fig. 1G). Together, these observations indicate that prominent abundance of T lymphocytes in sputum is associated with lower systemic inflammation and lower infection burden in pwCF.

### Coordinated changes in immune cell landscape, systemic inflammation and key clinical parameters during ETI treatment

**Fig. 2:**
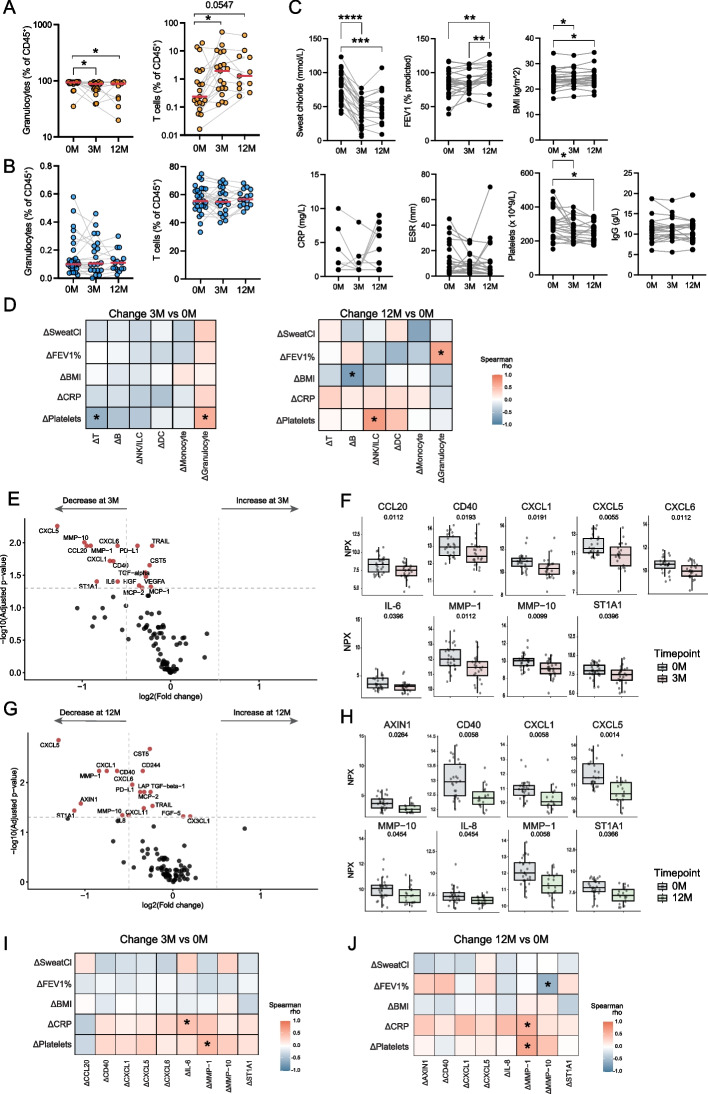
ETI treatment effects on immune cell compartments, clinical parameters and inflammatory markers. **A** Scatter dot plots of changes in T cell and granulocyte percentage out of CD45^+^ in the sputum of pwCF at baseline (0 M *n* = 21), three months (3 M *n* = 19) and 12 months (12 M *n* = 11) since ETI initiation. Scale is logarithmic (Wilcoxon test, *p < 0.05, **p < 0.01, ***p < 0.001). **B** Scatter dot plots of changes in T cell and granulocyte percentages out of CD45⁺ cells in the blood of pwCF at baseline (0 M *n* = 27), 3 months (3 M *n* = 20), and 12 months (12 M *n* = 15) since ETI treatment (Wilcoxon test, *p* < 0.05, *p < 0.01, **p < 0.001). **C** Scatter dot plots of clinically relevant parameters and their change during ETI. Sweat chloride concentration (mmol/L), FEV1 (% predicted), BMI (kg/m^2^), CRP (mg/L), ESR (mm), platelets (× 10^9^/L) and IgG levels (g/L) are depicted (0 M *n* = 25–27, 3 M *n* = 23–24, 12 M *n* = 18–20, varying per parameter) (Wilcoxon test, *p < 0.05, **p < 0.01). **D** Heatmap showing Spearman correlations between the change (Δ) in clinical parameters and change in cell percentages in the sputum after 3 months (*n* = 14–16, varying per parameter) and after 12 months (*n* = 8–10, varying per parameter) (*p < 0.05). **E** Volcano plot showing the log2 fold change of inflammatory plasma proteins at 3 months versus at baseline (Wilcoxon test with adjusted *p-*value < 0.05). A log2 fold change of 0.5 was set as cut-off (*n* = 23). **F** Boxplots showing the NPX values at baseline and 3 months after ETI of the top 11 proteins with an adjusted *p-*value < 0.05 and a log2 fold change > 0.5 (Wilcoxon test, adjusted *p-*values). **G** Volcano plot showing the log2 fold change of inflammatory plasma proteins at 12 months versus at baseline (Wilcoxon test with adjusted *p-*value < 0.05). A log2 fold change of 0.5 selected as cut-off (*n* = 17). **H** Boxplots showing the NPX values at baseline and 12 months after ETI of the top 7 proteins with an adjusted *p-*value < 0.05 and a log2 fold change > 0.5 (Wilcoxon test, adjusted *p-*values). **I** Heatmap showing Spearman correlations between change (Δ) in clinical parameters and change in inflammatory plasma proteins that have a fold change > 0.5 and an adjusted *p-*value < 0.05 after 3 months of ETI (*p < 0.05) (*n* = 21–23, varying per parameter). (**J**) Heatmap showing Spearman correlations between change (Δ) in clinical parameters and change in inflammatory plasma proteins that have a fold change > 0.5 and an adjusted *p-*value < 0.05 after 12 months of ETI (*p < 0.05) (*n* = 13–17, varying per parameter). Red line represents median in panels (**A**) and (**B**)

We next examined the impact of ETI therapy on cellular immune composition in sputum and peripheral blood, plasma inflammatory markers, and key clinical parameters. A significant reduction in the proportion of granulocytes in sputum was evident at three months of treatment and remained stable throughout the 12-month period (Fig. 2A). This was accompanied by a concomitant increase in T cell frequencies, suggesting a shift from a predominantly neutrophilic to a more lymphocyte-enriched environment. On the contrary, there were no changes in the granulocyte and T cell frequencies in the matching blood samples (Fig. 2B). Similarly, sputum B cell abundance increased at three months, while monocytes slightly decreased in the sputum between 3 and 12 months. NK/ILC and DC frequencies were not altered during ETI in either sputum or peripheral blood (Supplemental Fig. 2, A and B).

As expected, ETI promoted significant improvement in several important clinical parameters. Sweat chloride concentration, a biomarker of CFTR activity, decreased significantly within the first three months of treatment and remained low. Lung function assessed by FEV_1_ improved with slower kinetics with significant improvement at the 12-month timepoint. Body mass index, a general indicator of nutritional status, also increased slightly over time. Regarding systemic inflammation, platelet counts decreased during ETI treatment, indicating a reduction in inflammatory burden. In contrast, the ESR and serum total IgG levels remained unchanged. CRP levels were mostly relatively low at baseline and remained unchanged during ETI treatment (Fig. 2C). The decrease in platelet counts correlated positively with the decline in sputum granulocytes and was associated with the increase in T cell frequencies at 3 months. Interestingly, the increase in lung function correlated positively with the reduction in granulocyte frequencies in sputum after 12 months of ETI treatment (Fig. 2D). These findings support the notion that T cell enrichment in the airways may reflect a shift toward a less inflammatory and more balanced immune environment following ETI therapy. Additionally, this immunological shift was associated with favorable changes in clinical parameters, suggesting that the observed immunological effects may be clinically relevant.

Plasma proteome profiling revealed significant changes in the systemic inflammatory landscape following initiation of ETI treatment. Volcano plot analysis comparing baseline with the 3-month timepoint identified 9 proteins for which expression levels significantly decreased, with a fold change greater than 0.5 (Fig. 2E and F). These included a range of chemokines involved in immune cell recruitment such as CCL20, CXCL1, CXCL5, and CXCL6, as well as the inflammatory mediator, IL-6. In addition, matrix metalloproteinases MM*P-*1 and MM*P-*10, which are involved in tissue remodeling and inflammation, were significantly reduced. The co-stimulatory receptor CD40, and ST1A1 (sulfotransferase family 1 A member 1), an enzyme associated with acute inflammatory responses, were also downregulated (Fig. 2E and F). At the 12-month timepoint, several proteins remained significantly altered (Fig. 2, G and F). These included CD40, CXCL1, CXCL5, MM*P-*1, MM*P-*10, ST1A1as well as AXIN1 and the neutrophil attractant IL-8.

Decline of some inflammatory proteins correlated with clinical laboratory markers of systemic inflammation including CRP and platelet counts (Fig. 2I and J). Specifically, the change in IL-6 levels at three months (ΔIL-6) correlated positively with the corresponding change in CRP (ΔCRP). Furthermore, the decrease in MM*P-*1 levels showed consistent associations across both timepoints: at three months, the decrease correlated with the decrease in platelet counts, while at 12 months the decrease significantly correlated with both reduced CRP levels and platelet counts. These findings identify coordinated changes in immune cell landscape, systemic inflammation and key clinical parameters during ETI treatment, and reinforce the utility of plasma proteomics in capturing shifts in systemic inflammation following ETI treatment.

### ETI treatment modulates T cell subset distribution in both blood and sputum

**Fig. 3:**
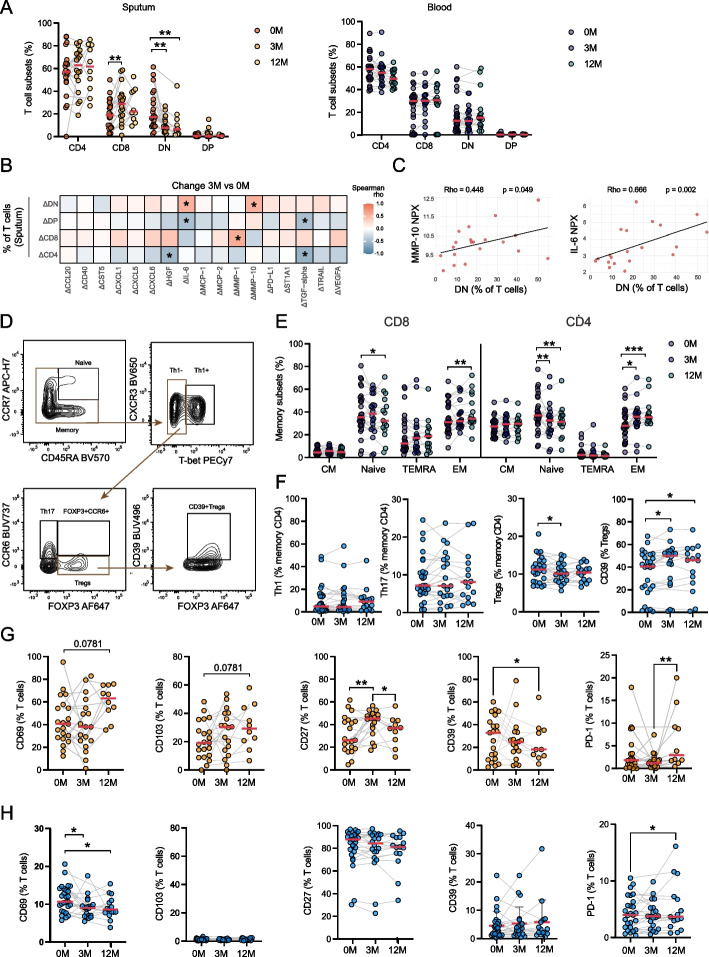
T cell compartment changes after three months and 12 months of ETI treatment. **A** Scatter dot plots showing changes in frequency of the CD4 and CD8 T cell compartments in sputum (0 M *n* = 21, 3 M *n* = 18, 12 M *n* = 10) and in the blood (0 M *n* = 27, 3 M *n* = 20, 12 M *n* = 15) of pwCF undergoing ETI treatment (Wilcoxon test, *p < 0.05, **p < 0.01). **B** Heatmap showing Spearman correlations between the change in the top 17 significant plasma proteins and the change in CD4 and CD8 T cells (%) in the sputum after 3 months of ETI (*p < 0.05, **p < 0.01) (*n* = 19). **C** Spearman correlations between the percentage of DN T cells in the sputum and the concentration of MM*P-*10 and IL-6 in the plasma of pwCF at baseline (*n* = 20). **D** Representative flow cytometry plots depicting the gating strategy of CD4 memory subsets. **E** Scatter dot plots depicting the main memory subsets in CD8 and CD4 T cells in blood during ETI treatment (0 M *n* = 27, 3 M *n* = 20, 12 M *n* = 15) (Wilcoxon test, *p < 0.05, **p < 0.01, ***p < 0.001, ****p < 0.0001). **F** Scatter dot plots showing CD4 T cell subset changes in blood during ETI (0 M *n* = 27, 3 M *n* = 20, 12 M *n* = 15) (Wilcoxon test, *p < 0.05). **G**,** H** Scatter dot plots showing the change in expression levels of surface markers on T cells. Shown are the percentages of CD69, CD103, CD27, CD39 and PD-1 on T cells in the sputum (0 M *n* = 21, 3 M *n* = 18, 12 M *n* = 10) (**G**) and blood (0 M *n* = 27, 3 M *n* = 20, 12 M *n* = 15) (**H**) during ETI treatment (Wilcoxon test, *p < 0.05, **p < 0.01, ***p < 0.001). Red line represents median in panels (**A**) and (**E–H**)

To investigate the effects of ETI treatment on the T cell compartment, we performed a longitudinal analysis of T cell subsets in both sputum and blood. In the sputum, ETI treatment led to a marked reduction in the frequency of CD4⁻CD8⁻ double negative (DN) T cells, accompanied by a corresponding increase in CD8⁺ T cells. These changes were only seen in sputum and remained stable over the 12-month period (Fig. 3A). Notably, the reduction in DN T cells correlated positively with the decrease in plasma levels of MM*P-*10 (ΔMM*P-*10) and IL-6 (ΔIL-6) after three months of treatment (Fig. 3B). Additionally, baseline frequencies of DN T cells in the sputum correlated positively with baseline plasma levels of both MM*P-*10 and IL-6, suggesting that this subset is associated with systemic markers of inflammation and may reflect disease-related immune dysregulation in CF that is improved by ETI therapy (Fig. 3C).

Overall, CD4⁺ and CD8⁺ T cell percentages were stable in peripheral blood over the course of treatment (Fig. 3A). However, analysis of the T cell memory subsets revealed a shift in both CD4⁺ and CD8⁺ T cells, with reduced naïve (CCR7^+^CD45RA^+^) cells, and a corresponding increase in effector memory (CCR7^−^CD45RA^−^) T (T_EM_) cells over 12 months of ETI treatment (Fig. 3D and E). Within the CD4⁺ memory T cell pool, there was a slight decrease in regulatory T cells (Tregs), accompanied by increased expression of the immunomodulatory ectonucleotidase CD39 on these cells (Fig. 3F). No changes were observed in the frequencies of T helper 1 (Th1) or T helper 17 (Th17) cells in peripheral blood throughout the follow-up period (Fig. 3, D-F). In addition, the frequency of Th17 at baseline was independent of the presence of chronic airway infection (Supplemental Fig. 3A).

Analysis of surface markers on T cells in sputum showed a trend towards an increase in the tissue-residency markers CD69 and CD103 after 12 months of ETI treatment, which could possibly suggest enhanced localization or retention of T cells within the airway mucosa (Fig. 3G). The co-stimulatory receptor CD27, commonly expressed on effector memory T cells, showed a transient increase in expression after three months of treatment; however, this effect was not sustained over time. CD39 and programmed cell death protein-1 (PD-1) also exhibited dynamic changes, although the pattern of modulation varied across individuals (Fig. 3G). In peripheral blood samples, expression of CD69 significantly decreased over the course of treatment, consistent with reduced systemic T cell activation as in blood CD69 is primarily viewed as an activation marker. Similarly, somewhat reduced PD-1 expression on circulating T cells suggested a shift toward a less activated or less exhausted phenotype on ETI therapy (Fig. 3H). Other markers of activation such as CD25, HLA-DR, CD38 and LAG-3 remained unaltered (Supplemental Fig. 3, B and C). Collectively, these findings indicate that ETI therapy modulates the T cell compartment with potential implications for the long-term benefits and safety of this treatment.

### Enhanced function of influenza virus-specific T cell responses during ETI treatment

**Fig. 4:**
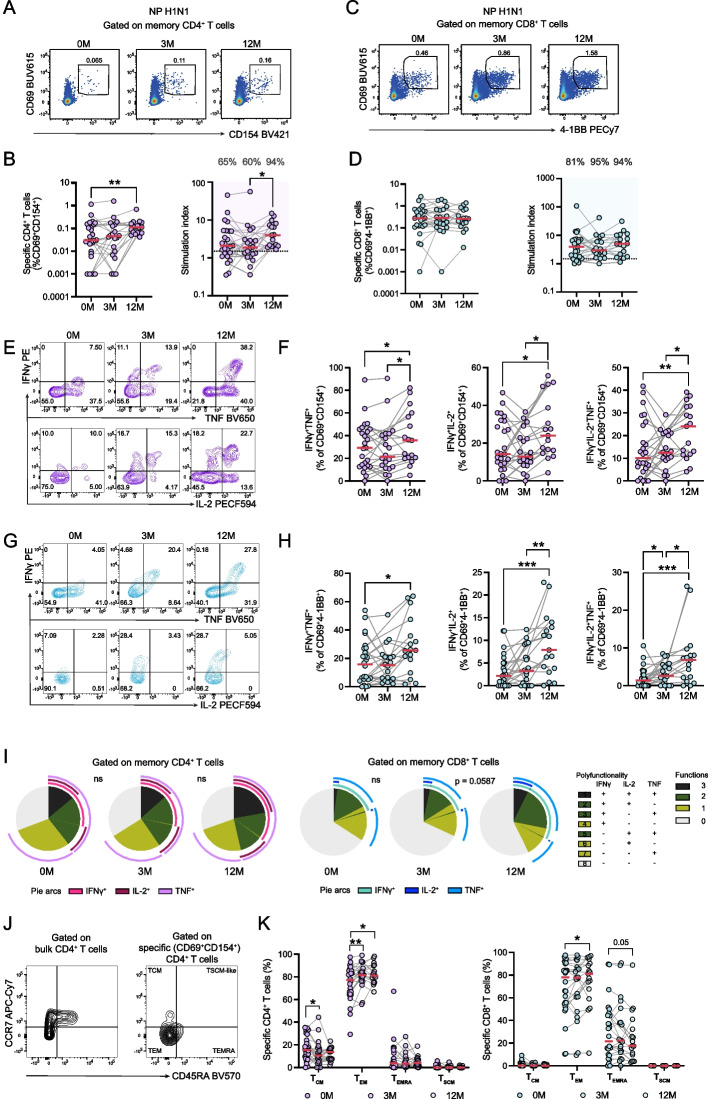
Dynamic enhancement of Influenza A specific T cell responses during ETI treatment. **A** Representative flow cytometry plots depicting the identification of influenza A virus (IAV)-specific CD4 T cell responses to H1N1 Nucleoprotein (NP) peptide pool in the blood of pwCF before the initiation and during ETI treatment. **B** Activation-induced marker (AIM) + memory CD4 T cells (left), and the stimulation index (fold-change) showing the percentage of responders (fold-change > 1.5) before (0 M), and during the course of ETI treatment at three months (3 M) and 12 months (12 M) (Wilcoxon test, *p < 0.05, **p < 0.01). **C** Representative flow cytometry plots depicting the identification of IAV-specific CD8 T cells in response to H1N1 Nucleoprotein (NP) peptide pool in the blood of pwCF before and during ETI treatment. **D** Frequencies of AIM + memory CD8 T cells (left), and the stimulation index (fold change) showing the responders (%) before and during ETI treatment. **E** Representative flow cytometry plots showing the cytokine expression in IAV-specific CD4 T cells measured by IFNγ, TNF and IL-2. **F** Scatter dot plots showing the quantification of cytokine expression in IAV-specific CD4 T cells (Wilcoxon test, *p < 0.05, **p < 0.01). **G** Representative flow cytometry plots showing the cytokine expression in IAV-specific CD8 T cells measured by IFNγ, TNF and IL-2. **H** Scatter dot plots showing the quantification of cytokine expression in IAV-specific CD8 T cells functional response (Wilcoxon test *p < 0.05, **p < 0.01, ***p < 0.001). **I** Pie charts depicting the polyfunctionality of AIM assay positive CD4 and CD8 T cells during ETI treatment (permutation test, ns = not significant). Pie arc coloring represents the molecule measured and pie chart coloring shows the number of functions (0 to 3). **J** Representative flow cytometry plot showing the gating strategy of T_CM_, T_EM_, T_EMRA_ and T_SCM_ among the IAV-specific CD4 T cells. **K** Scatter dot plots showing the representation of T_CM_, T_EM_, T_EMRA_ and T_SCM_ phenotypes within the IAV-specific CD4 and CD8 T cells (Wilcoxon test, *p < 0.05, **p < 0.01). For all analyses: 0 M *n* = 26, 3 M *n* = 20, 12 M *n* = 17. Red line represents median in all plots

To investigate the functional capacity of peripheral blood T cells during ETI treatment, we performed an activation-induced marker (AIM) assay using a nucleoprotein-derived peptide pool from IAV, an important viral lung pathogen. For memory CD4⁺ T cell responses, we quantified co-expression of CD69 and CD154 (CD40L) in response to stimulation (Fig. 4A and Supplemental Fig. 4A). Interestingly, there was a marked increase in the peptide-specific CD4⁺ T cell response to the IAV peptide pool at 12 months of ETI treatment (Fig. 4B). Memory CD8⁺ T cell responses were determined by co-expression of CD69 and 4-1BB (CD137) (Fig. 4C and Supplemental Fig. 4B). In contrast to the pattern observed with CD4⁺ T cells, memory CD8^+^ T cell responses to IAV were unchanged throughout the course of treatment (Fig. 4D). As an internal control, SEB was used as a potent stimulator to assess the response capacity of both CD4 and CD8 T cells to broad and non-specific stimulus and no change was observed during treatment for either subset (Supplemental Fig. 4, C and D).

To assess the capacity of IAV-specific CD4⁺ and CD8⁺ T cells to produce cytokines, we measured the expression of IFNγ, tumor necrosis factor (TNF), and IL-2 in response to peptide stimulation by intracellular flow cytometry. Notably, IAV-specific CD4⁺ T cells demonstrated gradually enhanced cytokine expression in response to antigen stimulation over the course of ETI treatment (Fig. 4E and F). Similarly, CD8⁺ T cells exhibited a robust increase in cytokine responses over time (Fig. 4G and H). In cytokine co-expression analyses, there was a significant increase in triple-positive IAV-specific cells especially between baseline and 12 months of ETI treatment for both CD4 and CD8 T cells (Fig. 4F and H), and an increase in overall polyfunctionality between the three- and 12-month timepoints observed for specific CD8^+^ T (Fig. 4I). Finally, analysis of memory subset characteristics, namely stem cell-like memory T (T_SCM_) cells (CCR7^+^CD45RA^+^), T_CM_ cells (CCR7^+^CD45RA^−^), T_EM_ cells (CCR7^−^CD45RA^−^), and effector memory RA^+^ T (T_EMRA_) cells (CCR7^−^CD45RA^+^) within the IAV-specific CD4^+^ T cell population (Fig. 4J), revealed a sustained shift towards T_EM_ characteristics accompanied by a decrease in T_CM_ characteristics throughout the treatment period. In contrast, the IAV-specific CD8⁺ T cell pool was largely stable in terms of memory subset characteristics over the 12 months of study (Fig. 4K).

Together, these findings indicate that in particular CD4^+^ T cell responses against IAV improve both in terms of magnitude and quality during ETI treatment.

### Increased MAIT cell numbers in sputum with retained functional responses to Pseudomonas aeruginosa

**Fig. 5:**
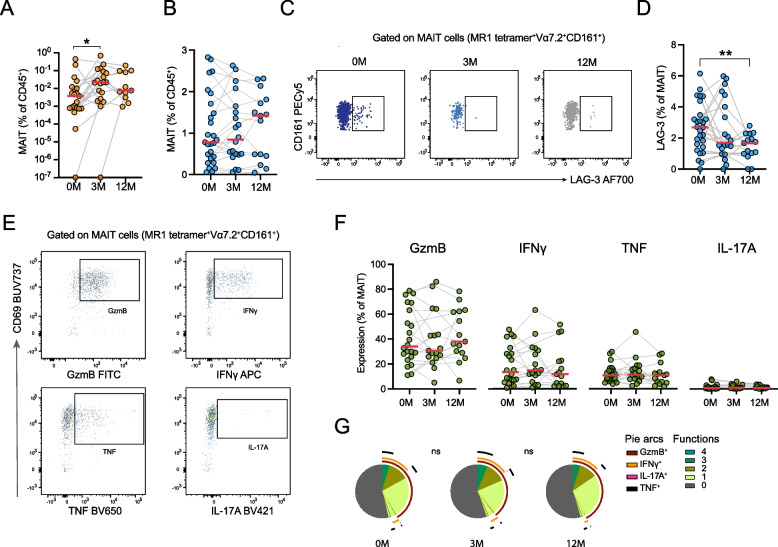
MAIT cell phenotype and function in individuals undergoing ETI treatment. **A** Scatter dot plot showing the MAIT cell percentages in the sputum of pwCF during ETI at baseline (0 M *n* = 21), three months (3 M *n* = 19) and 12 months (12 M *n* = 11). Scale is logarithmic (Wilcoxon test, *p < 0.05). **B** Scatter dot plot showing the MAIT cell percentages in the blood of pwCF during ETI (0 M *n* = 27, 3 M *n* = 20, 12 M *n* = 15). **C** Representative flow cytometry plots of LAG-3 expression on the surface of MAIT cells in the blood. **D** Scatter dot plot showing the expression of LAG-3 on MAIT cells in the blood during ETI (0 M *n* = 27, 3 M *n* = 20, 12 M *n* = 15) (Wilcoxon test, *p < 0.05, **p < 0.01, ***p < 0.001). **E** Representative flow cytometry plots of the MAIT cell response after stimulation with *P. aeruginosa* (MOI = 50) measured as expression of GzmB, IFNγ, TNF and IL-17A by intracellular flow cytometry. **F** Scatter dot plots showing the quantification of MAIT cell responses against *P. aeruginosa* (MOI = 50) during ETI treatment (0 M *n* = 22, 3 M *n* = 17, 12 M *n* = 15) (Wilcoxon test). **G** Pie charts depicting the polyfunctionality of MAIT cells responding to *P. aeruginosa* (MOI = 50) during ETI treatment (permutation test, ns = not significant). Pie arc coloring represents the functions measured and pie chart coloring shows the number of functions (0 to 4) (0 M *n* = 22, 3 M *n* = 17, 12 M *n* = 15). Red line represents median in panels (**A-B**), (**D**) and (**F**)

MAIT cells are unconventional T cells with an important role in immune defense of barrier tissues including the lung [32, 33]. MAIT cell frequencies in the sputum, but not in circulation, increased significantly during ETI treatment, mirroring the expansion observed in conventional T cells (Fig. 5A and B). However, characterization of MAIT cell phenotype in peripheral blood revealed reduced expression of the inhibitory molecule LAG-3 at 12 months after ETI initiation (Fig. 5C and D). Other activation and inhibitory markers, including CD38, CD25 and CD39 as well as the MAIT cell subset-defining marker CD56 [33], remained unchanged during treatment, although a trend towards reduced CD69 expression was observed (Supplemental Fig. 5A).

To assess the functional capacity of MAIT cells during ETI treatment, PBMCs were stimulated with mildly fixed *P. aeruginosa*, a common CF-associated pulmonary pathogen. Mild fixation allows antigen presentation while preventing further microbial growth. MAIT cell responses were evaluated by intracellular flow cytometry for the expression of granzyme B (GzmB), IFNγ, TNF, and IL-17A. MAIT cells were responsive upon stimulation with *P. aeruginosa* at baseline and responses remained stable throughout the course of treatment (Fig. 5E and F). The polyfunctional profile of MAIT cells, defined by the simultaneous expression of multiple effector molecules, remained unchanged during treatment, indicating that MAIT cell effector capacity was not significantly modulated by ETI treatment (Fig. 5G). Similar patterns of responses were observed for conventional non-MAIT T cells (Supplemental Fig. 5B-D).

## Discussion

The development of CFTR modulators has revolutionized CF care worldwide. ETI therapy markedly improves lung function and reduces pulmonary exacerbations in pwCF with at least one F508del allele [20, 21]. However, detailed investigations of the immunomodulatory effects of ETI treatment are needed to understand the broad spectrum of potential long-term benefits and side effects of the treatment, as well as to understand long-term-effects on lung infection dynamics and safety of ETI in clinically complex pwCF. Here, we investigated the immunological effects of ETI at the systemic level, using peripheral blood samples, and local pulmonary level, using non-invasive sputum sampling, in relation to key clinical parameters and sweat chloride concentrations. High-dimensional flow cytometry and plasma proteomics were employed to evaluate phenotypic and functional alterations in the immune system over a period of 12 months after ETI treatment initiation. The findings indicate that the T cell compartment is intimately linked with the clinical status and treatment outcomes, and that the quality of adaptive T cell immunity is improved.

The vicious cycle of airway infection and inflammation is a hallmark of CF. We found that key inflammatory mediators in plasma, including IL-8 and IL-17A, correlated negatively with T cell abundance in sputum before ETI treatment initiation. This was also the case for proteins indicative of tissue damage and repair, including HGF and TGFα. Thus, more abundant representation of T cells in sputum was associated with a healthier status. Interestingly, these associations were only seen for sputum T cells and not for their blood counterparts. In line with this, chronic infection by common CF-associated pathogens at baseline was associated with lower abundance of T cells and MAIT cells in sputum. Furthermore, individuals with chronic infection had elevated levels of unusual DN T cells within the sputum T cell pool, suggesting that reduced abundance of conventional CD4⁺ and CD8⁺ T cells is associated with higher prevalence of chronic infection. The importance of abundant T cells in sputum was further supported by the positive correlation between these cells and lower sweat chloride concentrations. All together, these findings support the notion that prominent abundance of T lymphocytes in sputum reflects lower grades of inflammation and lower prevalence of chronic infection in CF.

Previous studies have demonstrated the anti-inflammatory effects of ETI, with reduced inflammatory cytokine concentrations and normalization of neutrophil counts in circulation [23, 25, 34]. Consistent with these findings, our proteomics data revealed a profound reduction in inflammatory mediators in the plasma at three months post ETI initiation, and this pattern was at least partly sustained at 12 months. Systemic inflammation in CF is heavily influenced by IL-6, and the levels of this cytokine were reduced three months after treatment initiation. This change also correlated with a change in CRP levels, supporting the link between plasma IL-6 and hepatic acute phase responses [35]. The chemokine CCL20 (MI*P-*3α) is produced by macrophages and airway epithelium and attracts lymphocyte subsets and dendritic cells [36]. Previous studies have found elevated CCL20 levels in the bronchoalveolar lavage fluid of pwCF [37], with ETI modestly decreasing these levels [26, 38]. However, our data showed significant reduction of CCL20 after three months of ETI treatment, which could reflect a broader dampening of pro-inflammatory pathways as well as reduced epithelial activation, and its decline may serve as a marker of immune rebalancing. Metalloproteases MMP‑1 and MMP‑10 have been implicated in CF pathogenesis, with MMP‑1 exhibiting a well-characterized pro-inflammatory role and a reported association with impaired lung function [39, 40]. We found MM*P-*1 to be reduced in plasma three months after initiating treatment and that this effect was sustained at 12 months. The decline in MM*P-*1 levels was associated with decreased levels of platelets and CRP, highlighting its importance in the context of inflammation and tissue damage. IL-8 (CXCL8) mediates recruitment of neutrophils and levels of IL-8 are well known to be elevated in the sputum, bronchoalveolar lavage fluid and sera of children with CF [41]. A previous study indicated a modest decrease in IL-8 after six months of ETI, which was associated with improved lung function [26]. Here, we found that high plasma IL-8 concentration at baseline correlated with lower T cell abundance in the sputum, and ETI treatment significantly reduced IL-8 levels in plasma after twelve months of treatment. Other CXC chemokines that attract neutrophils, CXCL1 and CXCL5, were significantly reduced in the plasma after ETI initiation and remained reduced for the duration of the study.

Viral infections including those by IAV are a common trigger of lung exacerbations and respiratory symptoms in pwCF [17, 18, 42]. IAV also increases susceptibility to secondary bacterial infections, particularly *S. aureus* and *P. aeruginosa*, major drivers of recurrent pulmonary exacerbations and lung function decline in pwCF [43]. Notably, IAV has been shown to directly impair CFTR function and enhance bacterial infection in human bronchial epithelial cell models [44], underscoring the role of CFTR in modulating secondary infection risk. Here, we show that T cell responses to an IAV peptide pool antigen were significantly elevated in magnitude and exhibited greater polyfunctionality after ETI initiation. This was particularly clear for the CD4 T cell response to IAV antigen, which is important given the central role of adaptive CD4 T cells in immunity. These findings suggest that ETI therapy may offer protective effects beyond ion transport correction by limiting virus-induced vulnerability and secondary bacterial infections via a more effective adaptive immune response.

In addition to conventional adaptive T cell control of infections, unconventional T cells including MAIT cells are important for control of both bacterial and viral infections at barrier sites [32, 45, 46]. Here, we found that MAIT cell frequencies in the sputum increased during ETI, while remaining stable in the peripheral blood. Previously we described a unique case of a person with CF with severely impaired control of bacterial airway infections who was deficient in circulating MAIT cells, lending support to the hypothesis that MAIT cells may be crucial for infection control in the CF airways [47]. Thus, the increase in MAIT cell abundance in sputum during ETI might contribute to the decreased incidence of pulmonary exacerbations, which is a well-documented effect of ETI. Additionally, we recorded decreased MAIT cell surface expression of LAG-3 during ETI treatment. Consistent with our findings, a previous study reported an increase in MAIT cell numbers in blood three months after ETI initiation in CF children, which was accompanied by reduced LAG-3 expression [48]. In addition, elevated expression of LAG-3 on MAIT cells has been associated with increased hospitalization risk in patients with Chronic Obstructive Pulmonary Disease [49], suggesting that reduced LAG-3 expression following ETI treatment may reflect a disease-relevant pattern.

Chronic *P. aeruginosa* airway infection is very common in pwCF as the thick mucus, impaired immune response and bacterial immune evasion mechanisms allow for persistent biofilm formation. In the long run, this contributes to progressive lung damage. In this cohort, ETI therapy did not alter the response of peripheral blood MAIT cells or conventional T cells to *P. aeruginosa*. However, five out of eight chronically infected subjects cleared *P. aeruginosa* infection by 12 months after ETI initiation. In line with this, a considerable proportion of ETI-treated pwCF remains persistently infected with *P. aeruginosa* [50, 51]. All together, we speculate that other tools than CFTR modulators may be needed to eliminate *P. aeruginosa* infection in pwCF.

This study has several strengths, for example the longitudinal sampling of a real-world cohort, with paired sampling of sputum and blood, as well as the robust and detailed analysis of immune system dynamics. However, the study also has several limitations that should be mentioned. First, the patient cohort was heterogenous and comprised only 27 pwCF in total. Even though this contributed to the cohort’s representativeness, the study was not powered to study specific subgroups of patients. For example, it would have been interesting to compare the immunomodulatory impact of ETI between men and women with CF, subgroups with different microbiological status or genetic backgrounds, or to assess the influence of previous CFTR modulator treatment and antibiotics use. Second, the study was an observational real-world study, rather than a pure mechanistic experimental study. Thus, some of the patients discontinued ETI treatment before completing the 12-month follow-up and were excluded from sampling for immunological assays. Some received other simultaneous treatments as needed during the course of the study. Third, collection of sputum samples was not always possible, meaning we have slightly fewer data points from sputum analyses. Fourth, the long-term sustainability of the observed immunological effects is uncertain, as the follow-up period comprised 12 months only. Future studies should address long-term effects of ETI on inflammation and immunity. Lastly, we needed to freeze the PBMCs to allow simultaneous analysis of lymphocytes in longitudinal samples to avoid experimental variability. Therefore, the focus on flow cytometric analysis of PBMC did not allow precise assessment of granulocytes in blood.

## Conclusions

In summary, our findings indicate that ETI treatment induces both systemic and airway immune rebalancing, which aligns with significant clinical improvement in a real-world setting. T cells with lung resident characteristics, including MAIT cells, increase in sputum during ETI treatment, accompanied by improved lung function and reduced systemic inflammation. Furthermore, the peripheral blood effector-memory T cell pool expands and the quality of Influenza virus-specific CD4 and CD8 T cells improves. Thus, ETI treatment promotes remodelling of adaptive immunity in both blood and airways of pwCF that may contribute to improved clinical outcomes.

## Supplementary Information

Supplementary Material 1.

## Data Availability

All data generated or analysed during this study are included in this published article and its supplementary information files.

## Abbreviations

AIM: Activation-induced marker
BMI: Body mass index
CF: Cystic fibrosis
CRP: C-reactive protein
DC: Dendritic cell
DN: Double negative
ESR: Erythrocyte sedimentation rate
ETI: Elexacaftor/tezacaftor/ivacaftor
FEV₁: Forced expiratory volume in 1 s
GzmB: Granzyme B
HGF: Hepatocyte growth factor
IAV: Influenza A virus
MAIT: Mucosal-associated invariant T
NK: Natural killer
*P. aeruginosa*: *Pseudomonas aeruginosa*
PBMC: Peripheral blood mononuclear cells
PwCF: People with CF
SEB: Staphylococcal enterotoxin B
TGFα: Transforming growth factor α
TNF: Tumor necrosis factor
