# Supplementary material for: Systemic and airway T cell dynamics with influenza-specific immune recovery by cystic fibrosis elexacaftor/tezacaftor/ivacaftor therapy
Source: Respir Res. 2026 Jan 22;27:32. doi: 10.1186/s12931-026-03521-9 (PMC12849456; doi:10.1186/s12931-026-03521-9)
Supplement: Supplementary file 1 — Supplementary Material 1. [file 12931_2026_3521_MOESM1_ESM.pdf]

Supplementary material for the research article:

Systemic and airway T cell dynamics with influenza-specific immune recovery by cystic fibrosis elexacaftor/tezacaftor/ivacaftor therapy

Including 5 supplemental figures and 2 supplemental tables

Elli Mouchtaridi[1,a], Aleksandra Kowalik[2,3,a], Elisa JM Raineri[1], Marion Humbert[1], Josef Jägerstedt[2,3], Margaret Bojarlind[2,3], Kristina Nilsson[2,3], Malin Flodström-Tullberg[1], Terezia Pincikova[2,3,b], Johan K. Sandberg[1,b]

[1]Center for Infectious Medicine, Department of Medicine, Karolinska Institutet, Stockholm, Sweden. [2]Division of Pediatrics, Department of Clinical Science, Intervention and Technology, Karolinska Institutet, Stockholm, Sweden. [3]Stockholm CF Center, Karolinska University Hospital Huddinge, Stockholm, Sweden.

Address correspondence to: Dr. Johan K. Sandberg, CIM, Department of Medicine, Karolinska Institutet, Alfred Nobels Allé 8, 14152 Stockholm, Sweden. E-mail: johan.sandberg@ki.se; or to Dr. Terezia Pincikova, Stockholm CF Center, Albatross, K56, Karolinska University Hospital Huddinge, 14186 Stockholm, Sweden. E-mail: terezia.pincikova@ki.se

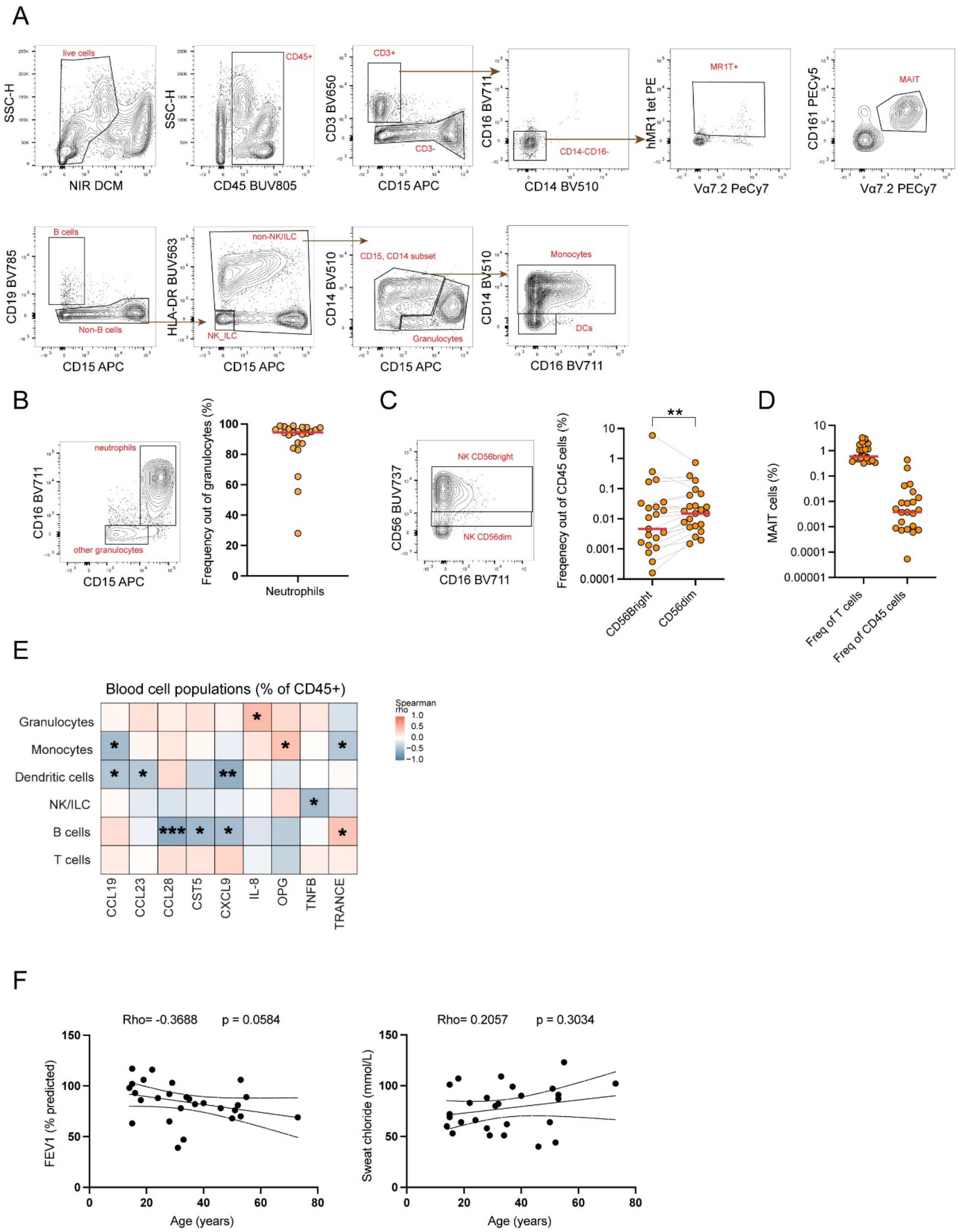

Supplemental Figure 1. Immune cell gating strategy and baseline correlations in sputum and blood. (**A**) Gating strategy for identification of major immune cell populations in sputum and blood. Representative plots from sputum samples shown. (**B**) Flow cytometry plot and scatter dot plot showing neutrophil percentages within the granulocyte population in sputum at baseline

(n=23). (**C**) Flow cytometry plot and scatter dot plot showing $CD56^{bright}$ and $CD56^{dim}$ NK cell percentages in $CD45^{+}$ sputum cells at baseline (n=23). Scale is logarithmic. (Wilcoxon test *p<0.05, **p<0.01). (**D**) Scatter dot plot showing MAIT cell percentages among total T cells and $CD45^{+}$ cells in sputum (n=23). Scale is logarithmic. (**E**) Heatmap of Spearman correlations between blood immune cell populations determined by flow cytometry and plasma proteome markers at baseline (n=26) (*p<0.05, **p<0.01, ***p<0.001). (**F**) Spearman correlation plots between age and FEV1 (% predicted) (left) and sweat chloride concentration (mmol/L) (right), at baseline (n=27). Red line represents median in panels (**B-D**).

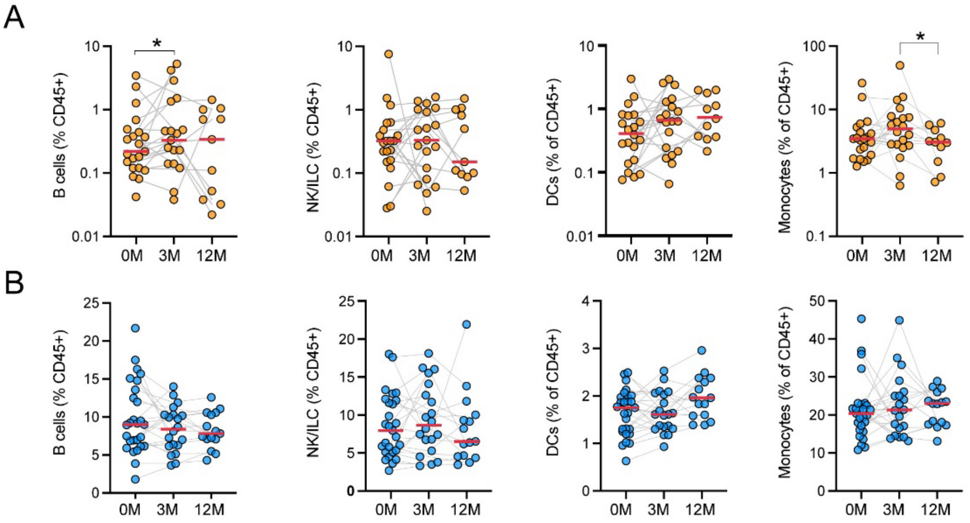

Supplemental Figure 2. ETI treatment effects on the immune cell compartment and correlations with clinically relevant parameters. (**A**) Scatter dot plots of changes in B cell, NK/ILC, DC and monocyte percentages in the sputum of pwCF during ETI (0M n=21, 3M n=19, 12M n=11). Scale is logarithmic (Wilcoxon test, *p<0.05). (**B**) Scatter dot plots of changes in B cell, NK/ILC, DC and monocyte percentages in the blood of pwCF during ETI (0M n=27, 3M n=20, 12M n=15). Red line represents median.

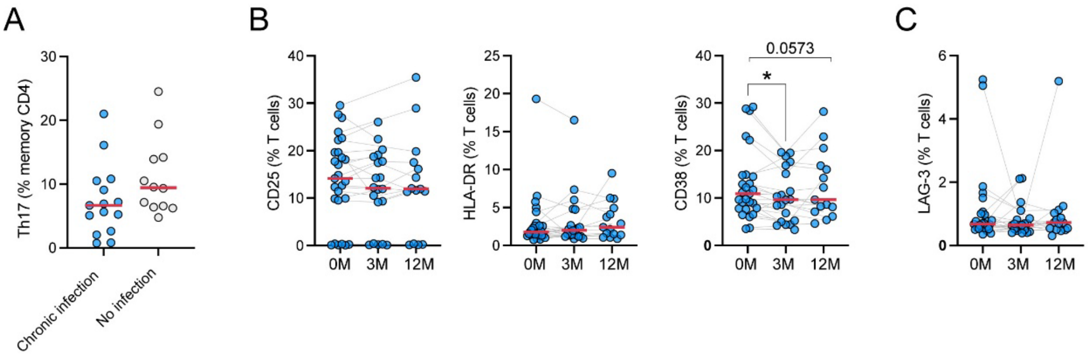

Supplemental Figure 3. **ETI treatment effects on peripheral blood T cell subsets and activation.** (**A**) Scatter dot plot showing baseline blood Th17 cell frequencies as percentage of memory CD4 T cells in pwCF with or without chronic infection (n=27) (Mann-Whitney test). (**B**) Scatter dot plots showing changes in T cell activation markers CD25, HLA-DR, and CD38 in T cells during ETI treatment (0M n=27, 3M n=20, 12M n=15) (Wilcoxon test, *$p<0.05$). (**C**) Scatter dot plot showing changes in LAG-3 expression in T cells during ETI treatment (0M n=27, 3M n=20, 12M n=15). Red line represents median.

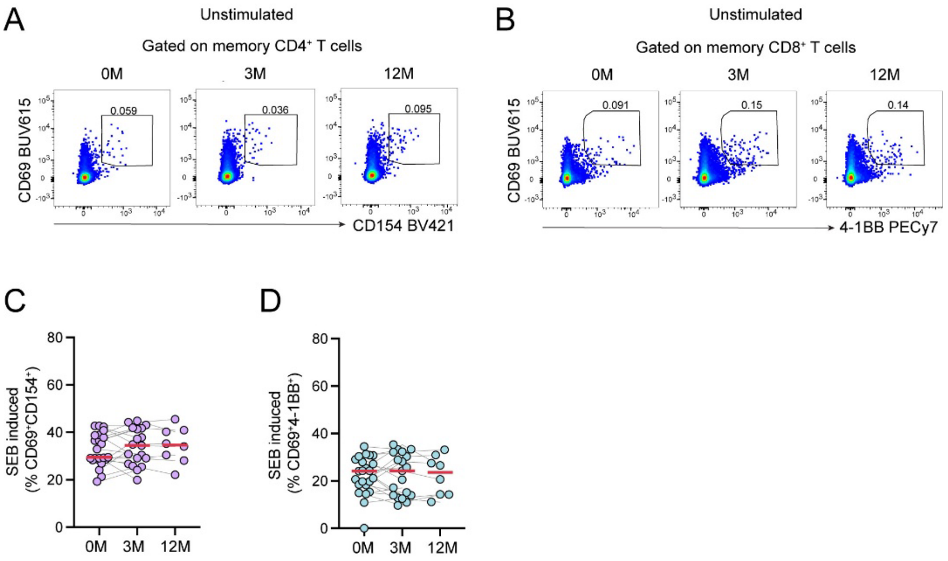

Supplemental Figure 4. Activation-induced marker (AIM) assay to assess antigen-specific T cell responses during ETI treatment. (**A**) Representative flow cytometry plots showing gating of IAV-specific CD4 T cells under unstimulated conditions at baseline (0M), 3 months (3M), and 12 months (12M) of ETI treatment. (**B**) Representative flow cytometry plots showing gating of IAV-specific CD8 T cells under unstimulated conditions at baseline, 3 months, and 12 months of ETI treatment. (**C**) Scatter dot plot showing SEB-induced CD4 T cell responses during ETI (0M n=23, 3M n=19, 12M n=8). **(D**) Scatter dot plot showing SEB-induced CD8 T cell responses during ETI 0M n=23, 3M n=19, 12M n=8). Red line represents median.

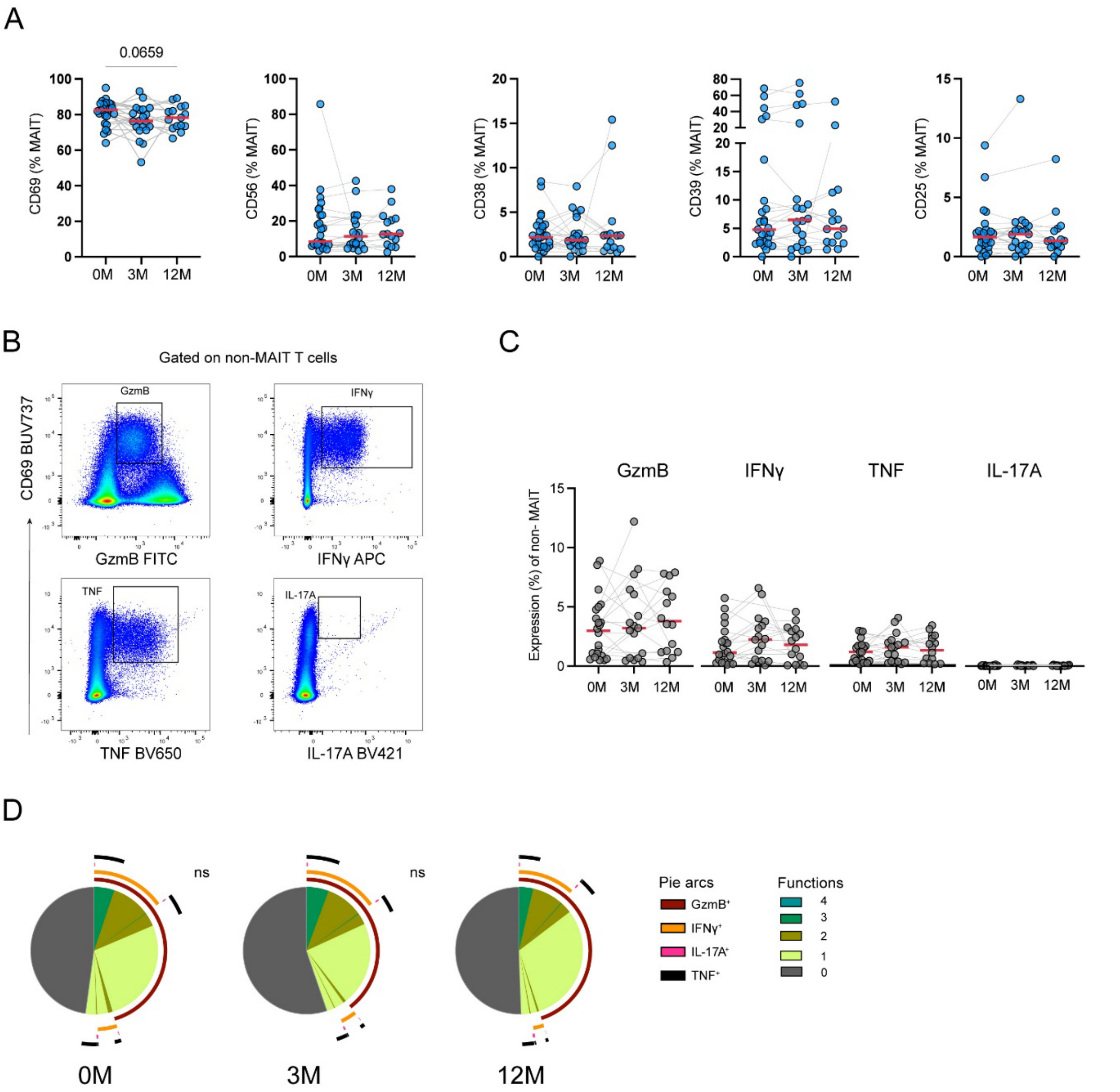

Supplemental Figure 5. MAIT cell phenotype and conventional T cell function following stimulation with *Pseudomonas* aeruginosa during ETI treatment. (**A**) Scatter dot plot showing changes in MAIT cell phenotypic marker expression in the blood of pwCF during ETI treatment (0M n=27, 3M n=20, 12M n=15) (Wilcoxon test *p<0.05). (**B**) Representative flow cytometry plots showing conventional non-MAIT T cell responses (GzmB, IFNγ, TNF, IL-17A) following stimulation with *Pseudomonas aeruginosa* (MOI=50). (**C**) Scatter dot plots quantifying conventional non-MAIT T cell responses to *Pseudomonas aeruginosa* (MOI=50) during ETI treatment (0M n=22, 3M n=17, 12M n=15) (Wilcoxon test, *p<0.05). (**D**) Pie charts depicting polyfunctionality of conventional T cells in response to *Pseudomonas aeruginosa* (MOI=50) during ETI treatment (permutation test, ns=non-significant). Pie arc colors represent cytokine markers. Pie coloring indicates number of functions (0-4) (0M n=22, 3M n=17, 12M n=15). Red line represents median in panels (**A**) and (**C**).

Supplemental Table 1. List of antibodies used for surface flow cytometry staining.

Antigen	Fluorochrome	Clone	Provider
CD3	BV650	OKT3	BioLegend
CD3	BUV805	SK7	BD Biosciences
CD8	BUV395	SK7	BD Biosciences
CD8	BV570	RPA-T8	BioLegend
CD4	BB700	SK3	BD Biosciences
Vα7.2	PE-Cy7	3C10	BioLegend
Vα7.2	APC	3C10	BioLegend
PD-1	FITC	EH12.2H7	BioLegend
PD-1	BV750	EH12.2H7	BioLegend
CD16	BV711	3G8	BioLegend
CD193	BV605	5E8	BioLegend
CD19	BV785	HIB19	BioLegend
CD19	V500	HIB19	BD Biosciences
CD14	V500	M5E2	BD Biosciences
CD15	APC	W6D3	BioLegend
CD161	PE-Cy5	DX10	BD Biosciences
CD103	BUV395	Ber-ACT8	BD Biosciences
CD69	AF700	FN50	BD Biosciences
CD69	BUV615	FN50	BD Biosciences
CD27	BUV615	M-T271	BD Biosciences
CD127	BUV661	HIL-7R-M21	BD Biosciences
CD39	BUV496	TU66	BD Biosciences
CD56	BUV737	NCAM16.2	BD Biosciences
CD45	BUV805	H130	BD Biosciences
CD62L	BV750	SK11	BD Biosciences
HLA-DR	BUV563	G46-6	BD Biosciences
CD38	BV421	HIT2	BioLegend
CD25	BV711	BC96	BioLegend
TIM-3	BV785	F38-2E2	BioLegend
LAG-3	AF700	T47-530	BD Biosciences
CTLA-4	PE/Dazzle594	BNI3	BioLegend
CD45RA	BV570	HI100	BioLegend
CCR6	BUV737	11A9	BD Biosciences
CCR7	APC-Cy7	G043H7	BioLegend
CXCR3	BV650	G025H7	BioLegend
CXCR5	BB515	RF8B2	BD Biosciences
Live/Dead Near IR	N/A	N/A	Invitrogen
Live/Dead AQUA	N/A	N/A	Invitrogen
5-OP-RU-hMR1	PE	N/A	NIH tetramer Core Facility

Supplemental Table 2. List of antibodies used for intracellular flow cytometry staining.

Antigen	Fluorochrome	Clone	Provider
IL-17A	BV605	BL168	BioLegend
IL-10	PE-CF594	JES3-19F1	BD Biosciences
IFNγ	PE	B27	BioLegend
Granzyme B	FITC	GB11	BioLegend
TNF	BV650	MAb11	BD Biosciences
IL-2	PE-CF594	5344.111	BD Biosciences
CD154	BV421	24-31	BioLegend
CD137	PE-Cy7	4B4-1	BioLegend
FOXP3	AF647	206D	BioLegend
TCF1/TCF7	AF488	C63D9	Cell Signaling
T-bet	PeCy7	eBio4B10	Invitrogen
